# A Practical Treatise on Impotence, Sterility and Allied Disorders of the Male Sexual Organs

**Published:** 1883-07

**Authors:** 


**A Practical Treatise on Impotence, Sterility and Allied Disorders of the Male Sexual Organs.** By SAMUEL W. GROSS, A. M., M. D., Professor of Surgery in Jefferson Medical College of Philadelphia. Second edition, thoroughly revised, with sixteen illustrations. Philadelphia: H. C. Lea's Son & Co. 1883.

This little book supplies, in a compact form, practical and strictly scientific information especially adapted to the wants of the general practitioner, in regard to a class of common and grave disorders upon the correction of which so much of human happiness depends. The writer shows a thorough familiarity with recent research into the scattered literature of this subject, and we regard the book as one of the highest value to the physician. It is excellently printed and sufficiently illustrated.

